# In Regards to Baubeta et al. JACMP 25(4), e14324

**DOI:** 10.1002/acm2.14429

**Published:** 2024-05-29

**Authors:** Leening P. Liu, Peter B. Noël

**Affiliations:** ^1^ Department of Radiology University of Pennsylvania Philadelphia Pennsylvania USA; ^2^ Department of Bioengineering University of Pennsylvania Philadelphia Pennsylvania USA

Dear Editor,

We carefully read a recently published paper by Baubeta et al., “No gadolinium k‐edge detected on the first clinical photon‐counting computed tomography scanner.” They aimed to characterize quantitative differences in gadolinium and iodinated contrast in photon‐counting CT (PCCT) to establish the feasibility of gadolinium k‐edge imaging.^1^ This topic is currently of high interest to the community, particularly following the recent clinical introduction of PCCT. However, we believe that the conclusion stating no gadolinium k‐edge was detected, and thus, gadolinium is not a clinically useful contrast agent for PCCT, is unfounded. The authors based their results on evaluations that, by definition, do not provide any insights into k‐edge imaging.

The paper highlights several technical flaws in the design and analysis of the experiments. However, the primary concern is the application of virtual monoenergetic images (VMI) in the context of k‐edge imaging. The authors utilized VMIs to attempt to establish differences in attenuation curves between iodine and gadolinium by visualizing the k‐edge of gadolinium at 50 keV. However, VMIs are based on a theoretical model in which an individual VMI level (keV) is constructed from two basis material maps generated through material decomposition. The principle underlying this two‐material decomposition is the two main x‐ray interactions in the diagnostic CT energy range: the photoelectric effect and Compton scattering. It fundamentally does not account for k‐edges, which additionally changes the energy dependence on attenuation for k‐edge materials. As a result, it is not unexpected that the authors did not observe the k‐edge of gadolinium in their attenuation curves from either PCCT or dual‐energy CT. For this reason, other studies on k‐edge imaging have adopted more sophisticated multi‐material decomposition techniques. These methods employ data from photon‐counting bins calibrated to the specific position of the k‐edge and integrate the unique energy dependence of a targeted k‐edge material as a separate basis to address its distinct characteristics.^2^, ^3^, ^4^ This would have been the appropriate method to evaluate whether PCCT can account for the k‐edge of gadolinium or any other suitable k‐edge‐based contrast agent.

In conclusion, the improper use of VMIs invalidates any conclusions drawn from the data regarding the feasibility of clinical k‐edge imaging with gadolinium. While the first clinical PCCT does not currently incorporate a clinical application of multi‐material decomposition or appropriate adjustment of the energy bin positions, the fundamental technology of this specific CT system, or photon‐counting detectors in general, does not prevent the performance of k‐edge imaging. The authors overlooked the necessary theoretical framework, and the practical experiments provided a limited contribution to the field. The conclusions drawn are not only invalid but unfortunately might also obstruct future clinical translation and applications of k‐edge imaging.

## CONFLICT OF INTEREST STATEMENT

Peter B. Noël has a research agreement with Canon Medical Systems Corporation, GE Healthcare, Philips Healthcare, and Siemens Healthineers. The other author does not have relevant conflicts of interest to disclose.
